# Impact of traditional Chinese medicine combined with conventional therapies on the survival of patients with metastatic prostate cancer: a retrospective observational cohort study

**DOI:** 10.3389/fonc.2026.1948429

**Published:** 2026-10-05

**Authors:** Jianfu Zhou, Shuyang Yang, Haoqiang Chen, Lei Hu, Yinglin Zhou, Hui Jiang, Jingmin Mai, Shu Gan, Zhiqiang Chen, Songtao Xiang

**Affiliations:** 1 Department of Urology, The Second Affiliated Hospital of Guangzhou University of Chinese Medicine, Guangzhou, China; 2 The Second Clinical College, Guangzhou University of Chinese Medicine, Guangzhou, China; 3 Department of Urology, The First Affiliated Hospital of Guangzhou University of Chinese Medicine, Guangzhou, China

**Keywords:** Cohort Studies, Medicine, Chinese Traditional, Neoplasm Metastasis, Proportional Hazards Models, Prostatic Neoplasms, Survival Analysis

## Abstract

**Background:**

Traditional Chinese medicine (TCM) is incorporated into the long-term integrative management of advanced prostate cancer in China, but evidence linking treatment duration with survival remains limited. Because treatment duration accumulates during follow-up, classifying patients by their final recorded treatment duration can misallocate pre-threshold observation time and bias survival comparisons.

**Methods:**

We evaluated the association between cumulative continuous TCM treatment exceeding 6 months and all-cause mortality in a single-center retrospective cohort of 242 patients with metastatic prostate adenocarcinoma at diagnosis who received continuous TCM from diagnosis. Follow-up continued through May 1, 2024. Treatment exceeding 6 months was modeled as a time-dependent exposure in Multivariable Cox regression, with sensitivity analyses addressing an alternative threshold, disease-severity adjustment, influential observations, and loss to follow-up.

**Results:**

During follow-up, 147 deaths occurred. Median overall survival was 53.0 months (95% confidence interval [CI], 44.0–61.0), with 3- and 5-year survival rates of 64.0% and 43.4%, respectively. Cumulative continuous TCM treatment exceeding 6 months was associated with a lower average hazard of all-cause mortality (adjusted hazard ratio [HR], 0.269; 95% CI, 0.186–0.389; P < 0.001). The association attenuated over time: adjusted HRs at 1, 2, 3, and 5 years were 0.098, 0.192, 0.286, and 0.471, respectively. Sensitivity analyses yielded estimates in the same direction.

**Conclusion:**

These findings define a duration-survival association among TCM users and support prospective, multicenter evaluation using detailed longitudinal treatment records.

## 1 Introduction

Prostate cancer is one of the most common malignancies and a leading cause of cancer death among men worldwide (1, 2). Its incidence and disease burden have increased in China, where late-stage presentation remains common and metastatic disease is associated with markedly poorer outcomes than localized disease (3–6). Androgen deprivation therapy (ADT) remains the foundation of systemic treatment for metastatic prostate cancer. Although most patients initially achieve disease control, endocrine resistance commonly develops and may culminate in metastatic castration-resistant disease, limiting long-term survival (7–10).

Novel androgen-receptor pathway inhibitors, chemotherapy, and ADT-based doublet or triplet regimens have improved survival in metastatic prostate cancer (10–16). However, patients treated in routine practice are often older and may have impaired performance status, multiple comorbidities, or limited tolerance of intensified systemic therapy; these factors are associated with lower uptake of treatment intensification in real-world cohorts (17). This heterogeneity underscores the need to evaluate adjunctive strategies in representative real-world populations across the disease course.

Traditional Chinese medicine (TCM) has a long history of use in the integrative management of prostate cancer and other malignancies in China (5). Its practice is based on syndrome differentiation and longitudinal adjustment to disease stage, physical condition, and treatment response (18–20). Preclinical studies and a recent review have reported that Chinese herbal compounds can inhibit prostate cancer growth and enhance the activity of antiandrogen treatments, including bicalutamide and enzalutamide (20–22). Clinical evidence, including a systematic review of randomized trials and a population-based retrospective cohort, has suggested that TCM combined with conventional treatment may delay disease progression and may be associated with longer survival, although the quality and scope of the evidence remain limited (23, 24). These findings provide a rationale for evaluating TCM within long-term, integrative management.

Treatment duration is an important but methodologically challenging dimension of long-term TCM exposure. Previous studies have generally compared users with non-users or classified patients at baseline according to their eventual treatment duration (23, 24). Because long-duration treatment is accumulated during follow-up, assigning the time before a patient reaches the duration threshold to the long-exposure group can introduce time-allocation bias (25, 26). We therefore modeled cumulative continuous TCM treatment exceeding 6 months as a time-dependent exposure in patients with metastatic prostate cancer who received TCM from diagnosis. Time-dependent Cox regression, time-varying effect estimation, Simon–Makuch curves, and prespecified sensitivity analyses were used to evaluate the association between treatment duration and all-cause mortality.

## 2 Materials and methods

### 2.1 Study design, setting, and ethics

This single-center retrospective observational cohort included patients treated at Guangdong Provincial Hospital of Chinese Medicine between January 2011 and December 2020. Follow-up information was updated through the data cutoff date of May 1, 2024. The study protocol was approved by the Ethics Committee of Guangdong Provincial Hospital of Chinese Medicine (approval no. YE2022-060-01) and registered with the Chinese Clinical Trial Registry (ChiCTR2200066332). The study was conducted in accordance with the Declaration of Helsinki. Because existing clinical records were used and some patients had died before data collection, the ethics committee waived the requirement for informed consent.

### 2.2 Patient selection and clinical definitions

All patients hospitalized with metastatic prostate cancer during the enrollment period were systematically screened against prespecified criteria. Patients were eligible if they were aged ≥18 years, had pathologically confirmed prostate adenocarcinoma, had radiologically confirmed distant metastasis (M1) at initial diagnosis, received active treatment, and had dates of initial diagnosis, survival status, and outcome ascertainment sufficient for survival analysis. Patients were excluded if they had prostate sarcoma, small-cell neuroendocrine carcinoma, another non-adenocarcinoma pathological subtype, or severe cardiovascular, cerebrovascular, renal, respiratory, or hematologic disease considered likely to materially affect survival.

Distant metastases were classified according to the eighth edition of the American Joint Committee on Cancer TNM system: M1a indicated non-regional lymph-node metastasis, M1b bone metastasis, and M1c other distant-organ metastasis with or without bone involvement (27). Tumor burden was classified using the CHAARTED criteria. High burden was defined as visceral metastasis or at least four bone lesions, including at least one lesion beyond the vertebral bodies and pelvis; all other patients were classified as having low burden (28). Disease risk was classified using the high-risk definition from the LATITUDE phase 3 trial in newly diagnosed metastatic castration-sensitive prostate cancer. High risk required at least two of the following: Gleason score ≥8, at least three bone lesions, or visceral metastasis; patients meeting no more than one criterion were classified as low risk (29).

### 2.3 Conventional treatment and TCM exposure

Conventional treatment was based on ADT and selected according to the patient’s clinical condition and the standards of care prevailing at the time. Treatment could include surgical or medical castration, first-generation antiandrogens, novel hormonal therapy, docetaxel chemotherapy, radiotherapy, and other appropriate systemic treatments. Conventional treatments were recorded as ever received.

TCM treatment primarily comprised individualized herbal decoctions and Chinese patent medicines prescribed according to syndrome differentiation. According to clinical confirmation by the research team, patients received continuous TCM treatment from their initial prostate cancer diagnosis. The database recorded each patient’s total duration of continuous TCM treatment; prescription-level dates, individual treatment start and stop dates, and interruptions were not available.

The primary exposure was a binary time-dependent variable indicating whether cumulative continuous TCM treatment had exceeded 6 months. Every patient was coded as unexposed to the >6-month state at diagnosis. For patients whose treatment duration exceeded 6 months, exposure was coded 0 from diagnosis until the 6-month threshold and 1 thereafter. Patients whose duration did not exceed 6 months remained coded 0 throughout follow-up. This approach assigned pre-threshold observation time to the ≤6-month state rather than retrospectively allocating it according to the patient’s eventual treatment duration.

### 2.4 Data collection

Trained research personnel extracted demographic, clinicopathological, treatment, and survival data from inpatient and outpatient electronic medical records, follow-up records, and related clinical documents. Baseline variables included age and year at initial diagnosis, ECOG PS, smoking history, comorbidities, baseline PSA, Gleason score, TNM stage, metastatic sites, tumor burden, and risk classification. Treatment variables included whether each conventional treatment had ever been received and the total duration of continuous TCM treatment.

Survival status and outcome dates were ascertained from inpatient and outpatient records, telephone follow-up, and available death-registration information. The date of death was recorded for deceased patients. For patients who remained alive or were lost to follow-up, the last date on which they were confirmed alive was recorded.

### 2.5 Outcome and follow-up

The primary outcome was OS, defined as the interval from the date of initial prostate cancer diagnosis to death from any cause. Follow-up began at diagnosis and ended at death or the last date the patient was known to be alive. Patients alive at the end of follow-up and those lost to follow-up were right-censored on their last confirmed-alive date.

### 2.6 Statistical analysis

Continuous variables were described using the mean and standard deviation or the median and IQR, according to their distribution. Categorical variables were summarized as counts and percentages. Baseline characteristics were described for the full cohort and, as a supplementary descriptive comparison, according to the final recorded duration of continuous TCM treatment. Between-group differences were expressed as absolute SMDs (30). Because baseline PSA was right-skewed and some values were recorded as thresholds rather than exact measurements, Cox models included the log2-transformed PSA lower bound and an indicator for inexact PSA recording.

The Kaplan–Meier method was used to estimate OS for the overall cohort, including median OS with its 95% CI and the 3- and 5-year survival rates. Individual observed time was measured from diagnosis to death or right censoring. Potential follow-up was estimated using the reverse Kaplan–Meier method (31).

The association between cumulative continuous TCM treatment exceeding 6 months and all-cause mortality was evaluated using time-dependent Cox regression in start–stop format. All patients entered with exposure status 0, and eligible patients changed to status 1 after exceeding the 6-month threshold. The prespecified multivariable model included age at diagnosis, year of diagnosis, ECOG PS (0–1 vs 2), log2-transformed baseline PSA lower bound, inexact PSA recording, Gleason score, and CHAARTED tumor burden. Tied event times were handled using the Efron method. Robust sandwich standard errors were clustered by patient.

The proportional hazards assumption was assessed using scaled Schoenfeld residuals (32). When the assumption was not satisfied for the primary time-dependent exposure, an extended Cox model was fitted with an interaction between the exposure and log(time/365). This model was used to estimate time-specific HRs and 95% CIs at 1, 2, 3, and 5 years after diagnosis. Simon–Makuch curves described survival experience across the current time-dependent exposure states and were not used as the primary effect estimate (33).

For methodological comparison, fixed Cox models classified patients according to their final recorded continuous TCM treatment duration (≤6 vs >6 months). Four prespecified sensitivity analyses were conducted: exclusion of patients with inexact baseline PSA records; replacement of CHAARTED tumor burden with LATITUDE risk classification; replacement of CHAARTED tumor burden with M1c status; and use of a 3-month rather than 6-month time-dependent exposure threshold. Unless otherwise specified, the models retained the covariates, tie handling, and robust standard-error estimation used in the primary analysis.

Patient-level standardized DFBETA values were used to assess individual influence on the time-dependent TCM exposure coefficient. Patients with an absolute standardized DFBETA greater than 2/√N were considered potentially influential (N = 242; cutoff, 0.1286) (34, 35). The full primary model was refitted after removing each flagged patient separately and after cumulatively excluding the one, three, and five most influential patients.

Patients lost to follow-up were right-censored on the last date they were confirmed alive. Sensitivity analyses first repeated the primary time-dependent Cox analysis after excluding all patients lost to follow-up. IPCW was then used to evaluate selective censoring explained by prespecified baseline covariates (36, 37). The censoring model included age, year of diagnosis, ECOG PS, log2-transformed baseline PSA lower bound, inexact PSA recording, Gleason score, and CHAARTED tumor burden. Results were reported using untruncated stabilized weights and weights truncated at the 1st and 99th percentiles. This analysis does not control for selective censoring caused by unmeasured factors.

Analyses were performed using R version 4.5.1. Time-dependent Cox models were fitted using the survival package version 3.8-3, and the proportional hazards assumption was assessed using the cox.zph() function. Data processing and visualization used appropriate R packages, including ggplot2. All tests were two-sided, and P<0.05 was considered statistically significant.

## 3 Results

### 3.1 Cohort characteristics

Between January 2011 and December 2020, 242 patients with distant metastases at their initial prostate cancer diagnosis met the eligibility criteria. The median age at diagnosis was 73.5 years (interquartile range [IQR], 66.0–79.0), and 158 patients (65.3%) were aged 70 years or older. The median year of diagnosis was 2017 (range, 2011–2020). Eastern Cooperative Oncology Group performance status (ECOG PS) was 0–1 in 203 patients (83.9%) and 2 in 39 (16.1%).

Hypertension was present in 111 patients (45.9%), diabetes mellitus in 42 (17.4%), and a history of current or previous smoking in 87 (36.0%). Median baseline prostate-specific antigen (PSA) lower-bound concentration was 185.0 ng/mL (IQR, 98.2–689.7). Gleason scores were ≤7 in 54 patients (22.3%), 8 in 98 (40.5%), and ≥9 in 90 (37.2%). A total of 129 patients (53.3%) had clinical T4 disease and 166 (68.6%) had N1 disease. M1a, M1b, and M1c disease was present in 53 (21.9%), 166 (68.6%), and 23 (9.5%) patients, respectively. According to the CHAARTED and LATITUDE criteria, 132 patients (54.5%) had high tumor burden and 163 (67.4%) had high-risk disease.

For descriptive purposes, 146 patients (60.3%) had a final recorded continuous TCM treatment duration of ≤6 months and 96 (39.7%) had a duration of >6 months. Absolute standardized mean differences (SMDs) exceeded 0.20 for baseline PSA (0.316), clinical T stage (0.232), CHAARTED tumor burden (0.222), and Gleason score (0.218); all other baseline variables had absolute SMDs below 0.20 (**Table 1**).

**Table 1:** Patient characteristics according to continuous TCM treatment duration.

Characteristic	Overall cohort (N = 242)	TCM duration ≤6 months (n=146)	TCM duration >6 months (n=96)	Absolute SMD
Age at diagnosis, years, median (IQR)	73.5 (66.0–79.0)	74.5 (66.0–79.0)	71.5 (66.0–79.0)	0.051
Age group, years				0.061
<70	84 (34.7%)	49 (33.6%)	35 (36.5%)	
≥70	158 (65.3%)	97 (66.4%)	61 (63.5%)	
Year of diagnosis, median (range)	2017 (2011–2020)	2016 (2011–2020)	2017 (2011–2020)	0.023
ECOG performance status				0.071
0–1	203 (83.9%)	124 (84.9%)	79 (82.3%)	
2	39 (16.1%)	22 (15.1%)	17 (17.7%)	
Hypertension				0.138
No	131 (54.1%)	83 (56.8%)	48 (50.0%)	
Yes	111 (45.9%)	63 (43.2%)	48 (50.0%)	
Diabetes mellitus				0.012
No	199 (82.6%)	120 (82.8%)	79 (82.3%)	
Yes	42 (17.4%)	25 (17.2%)	17 (17.7%)	
Missing	1 (0.4%)	1 (0.7%)	0 (0.0%)	
Smoking history				0.091
Never	155 (64.0%)	91 (62.3%)	64 (66.7%)	
Ever	87 (36.0%)	55 (37.7%)	32 (33.3%)	
Baseline PSA lower bound, ng/mL, median (IQR)	185.0 (98.2–689.7)	226.5 (100.0–1000.0)	100.3 (78.3–538.2)	0.316
Gleason score				0.218
≤7	54 (22.3%)	29 (19.9%)	25 (26.0%)	
8	98 (40.5%)	65 (44.5%)	33 (34.4%)	
≥9	90 (37.2%)	52 (35.6%)	38 (39.6%)	
Clinical T stage				0.232
≤T3a	65 (26.9%)	36 (24.7%)	29 (30.2%)	
T3b	48 (19.8%)	34 (23.3%)	14 (14.6%)	
T4	129 (53.3%)	76 (52.1%)	53 (55.2%)	
Clinical N stage				0.074
N0	51 (21.1%)	32 (21.9%)	19 (19.8%)	
N1	166 (68.6%)	100 (68.5%)	66 (68.8%)	
Nx	25 (10.3%)	14 (9.6%)	11 (11.5%)	
M stage				0.175
M1a	53 (21.9%)	36 (24.7%)	17 (17.7%)	
M1b	166 (68.6%)	96 (65.8%)	70 (72.9%)	
M1c	23 (9.5%)	14 (9.6%)	9 (9.4%)	
CHAARTED tumor burden				0.222
Low	110 (45.5%)	60 (41.1%)	50 (52.1%)	
High	132 (54.5%)	86 (58.9%)	46 (47.9%)	
LATITUDE risk classification				0.098
Low	79 (32.6%)	45 (30.8%)	34 (35.4%)	
High	163 (67.4%)	101 (69.2%)	62 (64.6%)	

SMDs are presented as absolute values. For continuous variables, SMDs were calculated using the original continuous measurements; for categorical variables, the reported value represents the overall standardized difference across categories. Patients were descriptively stratified according to their final recorded continuous TCM treatment duration. ECOG, Eastern Cooperative Oncology Group; IQR, interquartile range; PSA, prostate-specific antigen; SMD, standardized mean difference; TCM, traditional Chinese medicine.

SMDs are presented as absolute values. For continuous variables, SMDs were calculated using the original continuous measurements; for categorical variables, the reported value represents the overall standardized difference across categories. Patients were descriptively stratified according to their final recorded continuous TCM treatment duration. ECOG, Eastern Cooperative Oncology Group; IQR, interquartile range; PSA, prostate-specific antigen; SMD, standardized mean difference; TCM, traditional Chinese medicine.

### 3.2 Follow-up and overall survival

Among the 242 patients, 147 all-cause deaths (60.7%) were observed. The remaining 95 patients were right-censored, including 47 (19.4%) who were confirmed alive at their last follow-up and 48 (19.8%) who were lost to follow-up. Patients lost to follow-up were censored on the last date they were known to be alive.

The median observed time from diagnosis to death or censoring was 36.0 months. Median potential follow-up estimated using the reverse Kaplan–Meier method was 64.0 months. Median overall survival (OS) was 53.0 months (95% confidence interval [CI], 44.0–61.0), and the 3- and 5-year OS rates were 64.0% and 43.4%, respectively (**Figure 1**).

**Figure 1:**
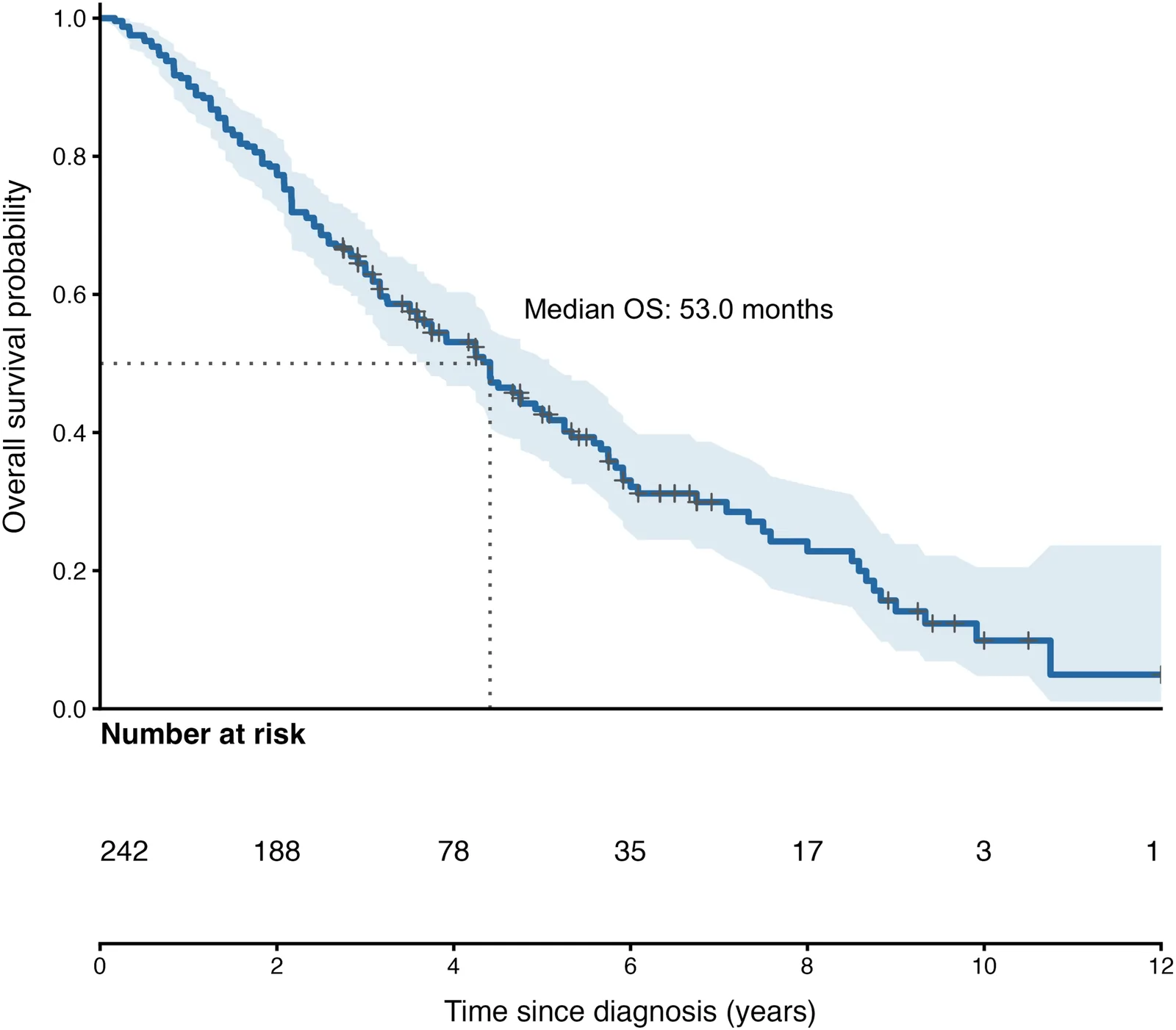
Overall survival of patients with metastatic prostate cancer. The Kaplan–Meier curve includes all available follow-up from diagnosis (n=242; 147 deaths). Shading represents the 95% confidence interval, and plus signs indicate censoring. Median overall survival was 53.0 months (95% CI, 44.0–61.0). Numbers below the plot show patients at risk at 0, 2, 4, 6, 8, 10, and 12 years.

The Kaplan–Meier curve includes all available follow-up from diagnosis (n=242; 147 deaths). Shading represents the 95% confidence interval, and plus signs indicate censoring. Median overall survival was 53.0 months (95% CI, 44.0–61.0). Numbers below the plot show patients at risk at 0, 2, 4, 6, 8, 10, and 12 years.

### 3.3 Association between cumulative continuous TCM treatment exceeding 6 months and all-cause mortality

In the primary multivariable time-dependent Cox model, cumulative continuous TCM treatment exceeding 6 months was associated with a lower hazard of all-cause mortality after adjustment for age at diagnosis, year of diagnosis, ECOG PS, log2-transformed baseline PSA lower bound, inexact PSA recording, Gleason score, and CHAARTED tumor burden (adjusted hazard ratio [HR], 0.269; 95% CI, 0.186–0.389; P<0.001). This estimate represents the average association over follow-up rather than a constant effect (**Table 2**). The complete time-varying estimates are reported in **Figure 2**; **Supplementary Table S1**.

**Table 2:** Multivariable time-dependent Cox regression analysis of all-cause mortality.

Variable	Adjusted HR	95% CI	P value
Time-dependent cumulative continuous TCM treatment >6 months	0.269	0.186–0.389	<0.001
Age, per 1-year increase	1.032	1.009–1.055	0.006
Year of diagnosis, per 1-year increase	0.963	0.908–1.022	0.213
ECOG performance status 2 (vs 0–1)	0.553	0.327–0.936	0.027
Baseline PSA lower bound, per two-fold increase	1.042	0.954–1.137	0.359
Inexact PSA record	0.861	0.536–1.384	0.538
Gleason score 8 (vs ≤7)	0.967	0.603–1.549	0.888
Gleason score ≥9 (vs ≤7)	1.341	0.843–2.131	0.215
High CHAARTED tumor burden (vs low)	1.347	0.940–1.929	0.104

n=242; deaths=147; 338 start–stop intervals. Tied event times were handled using the Efron method, and robust standard errors were clustered at the patient level. Baseline PSA was modeled as the log2-transformed PSA lower bound together with an indicator for inexact PSA recording. Because the proportional hazards assumption was not satisfied for the time-dependent TCM exposure, the reported HR represents an average association over follow-up and should not be interpreted as a constant effect. Time-specific estimates are presented in **Figure 2**; **Supplementary Table S1**. CI, confidence interval; ECOG, Eastern Cooperative Oncology Group; HR, hazard ratio; PSA, prostate-specific antigen; TCM, traditional Chinese medicine.

**Figure 2:**
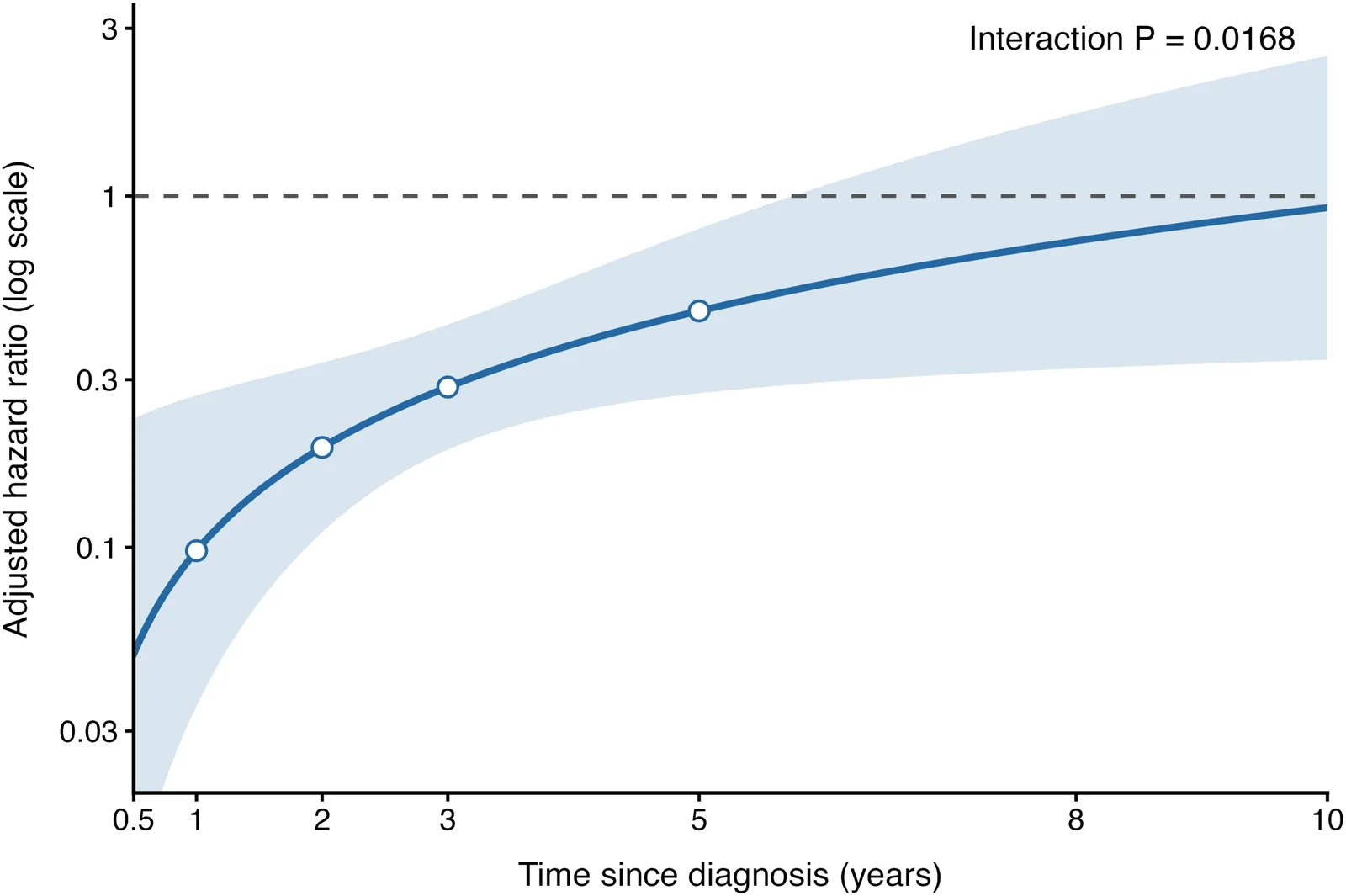
Time-varying association between cumulative continuous TCM treatment exceeding 6 months and all-cause mortality. The solid line represents the time-varying adjusted HR estimated from a Cox model containing an interaction between the time-dependent TCM exposure and log(time/365); the shaded area represents the 95% confidence interval. Open circles indicate estimated HRs at 1, 2, 3, and 5 years after diagnosis. The horizontal dashed line indicates an HR of 1. The curve begins at 0.5 years because the >6-month exposure state could not occur before the 6-month threshold. The interaction between TCM exposure and time was statistically significant (P = 0.0168). The model was adjusted for age at diagnosis, year of diagnosis, ECOG PS, log2-transformed baseline PSA lower bound, inexact PSA recording, Gleason score, and CHAARTED tumor burden. CI, confidence interval; ECOG, Eastern Cooperative Oncology Group; HR, hazard ratio; PSA, prostate-specific antigen; TCM, traditional Chinese medicine.

The solid line represents the time-varying adjusted HR estimated from a Cox model containing an interaction between the time-dependent TCM exposure and log(time/365); the shaded area represents the 95% confidence interval. Open circles indicate estimated HRs at 1, 2, 3, and 5 years after diagnosis. The horizontal dashed line indicates an HR of 1. The curve begins at 0.5 years because the >6-month exposure state could not occur before the 6-month threshold. The interaction between TCM exposure and time was statistically significant (P = 0.0168). The model was adjusted for age at diagnosis, year of diagnosis, ECOG PS, log2-transformed baseline PSA lower bound, inexact PSA recording, Gleason score, and CHAARTED tumor burden. CI, confidence interval; ECOG, Eastern Cooperative Oncology Group; HR, hazard ratio; PSA, prostate-specific antigen; TCM, traditional Chinese medicine.

n=242; deaths=147; 338 start–stop intervals. Tied event times were handled using the Efron method, and robust standard errors were clustered at the patient level. Baseline PSA was modeled as the log2-transformed PSA lower bound together with an indicator for inexact PSA recording. Because the proportional hazards assumption was not satisfied for the time-dependent TCM exposure, the reported HR represents an average association over follow-up and should not be interpreted as a constant effect. Time-specific estimates are presented in **Figure 2**; **Supplementary Table S1**. CI, confidence interval; ECOG, Eastern Cooperative Oncology Group; HR, hazard ratio; PSA, prostate-specific antigen; TCM, traditional Chinese medicine.

### 3.4 Proportional hazards assumption and time-varying association

The scaled Schoenfeld residual test indicated that the proportional hazards assumption was not satisfied for the time-dependent TCM exposure (P = 0.034; **Figure 3**). In the extended Cox model containing an interaction between the time-dependent exposure and log(time/365), the interaction was statistically significant (P = 0.017). The magnitude of the association decreased with time. Adjusted HRs at 1, 2, 3, and 5 years after diagnosis were 0.098 (95% CI, 0.035–0.270), 0.192 (95% CI, 0.110–0.335), 0.286 (95% CI, 0.190–0.431), and 0.471 (95% CI, 0.274–0.807), respectively. Each estimate was below 1, but the HR progressively approached the null value (**Figure 2**; **Supplementary Table S1**).

**Figure 3:**
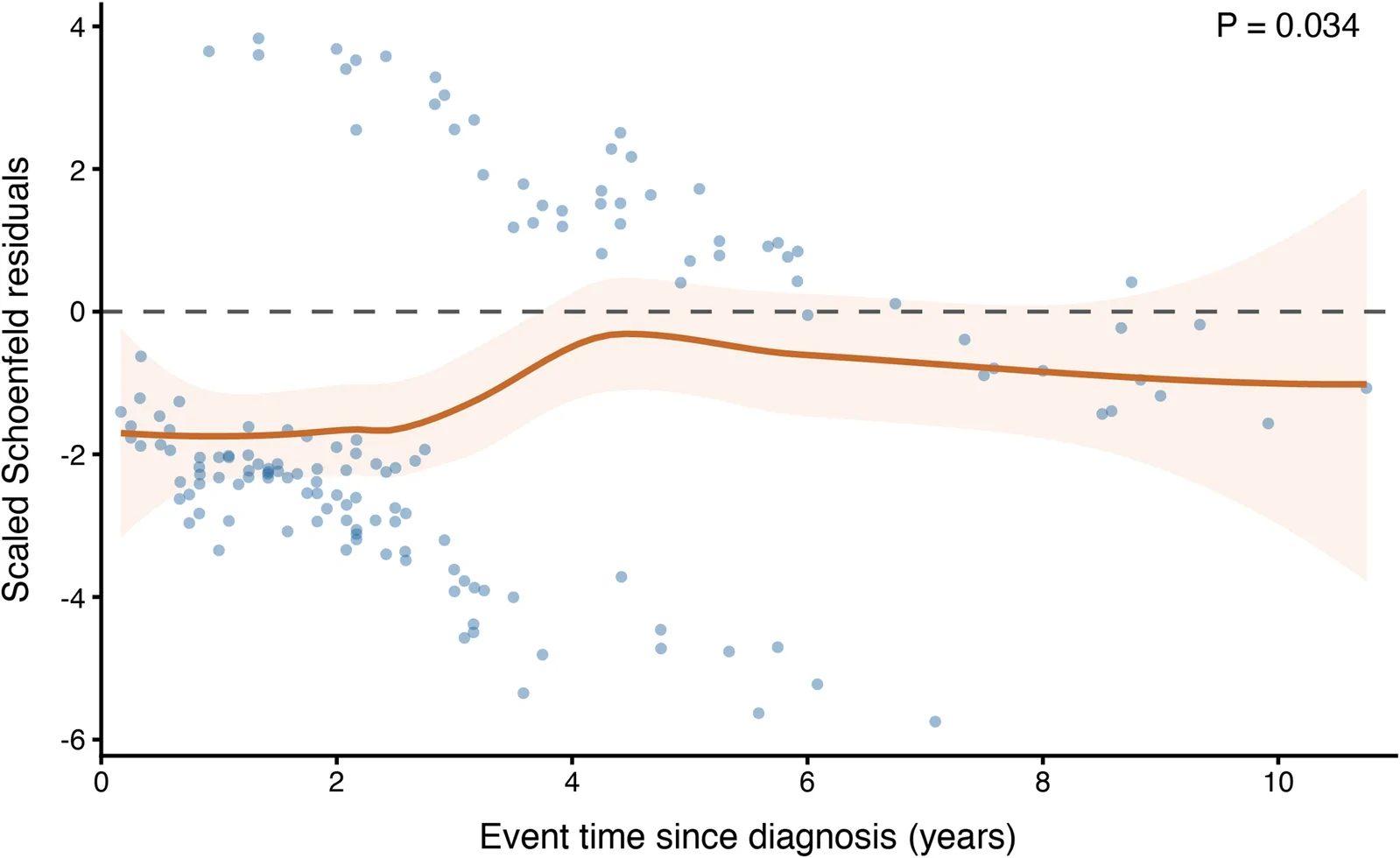
Scaled Schoenfeld residuals for the time-dependent cumulative continuous TCM treatment exposure. The smoothed residual curve and its 95% confidence interval indicate deviation from the proportional hazards assumption for the time-dependent TCM exposure (P = 0.034).

The smoothed residual curve and its 95% confidence interval indicate deviation from the proportional hazards assumption for the time-dependent TCM exposure (P = 0.034).

### 3.5 Descriptive survival curves for time-dependent exposure states

All patients entered follow-up in the cumulative continuous TCM treatment ≤6-month state. Ninety-six patients transitioned to the >6-month state after exceeding the 6-month threshold. For most of follow-up, the estimated survival probability in the >6-month state was higher than that in the ≤6-month state (**Figure 4**). The Simon–Makuch curves were descriptive; effect estimation was based on the time-dependent Cox model.

**Figure 4:**
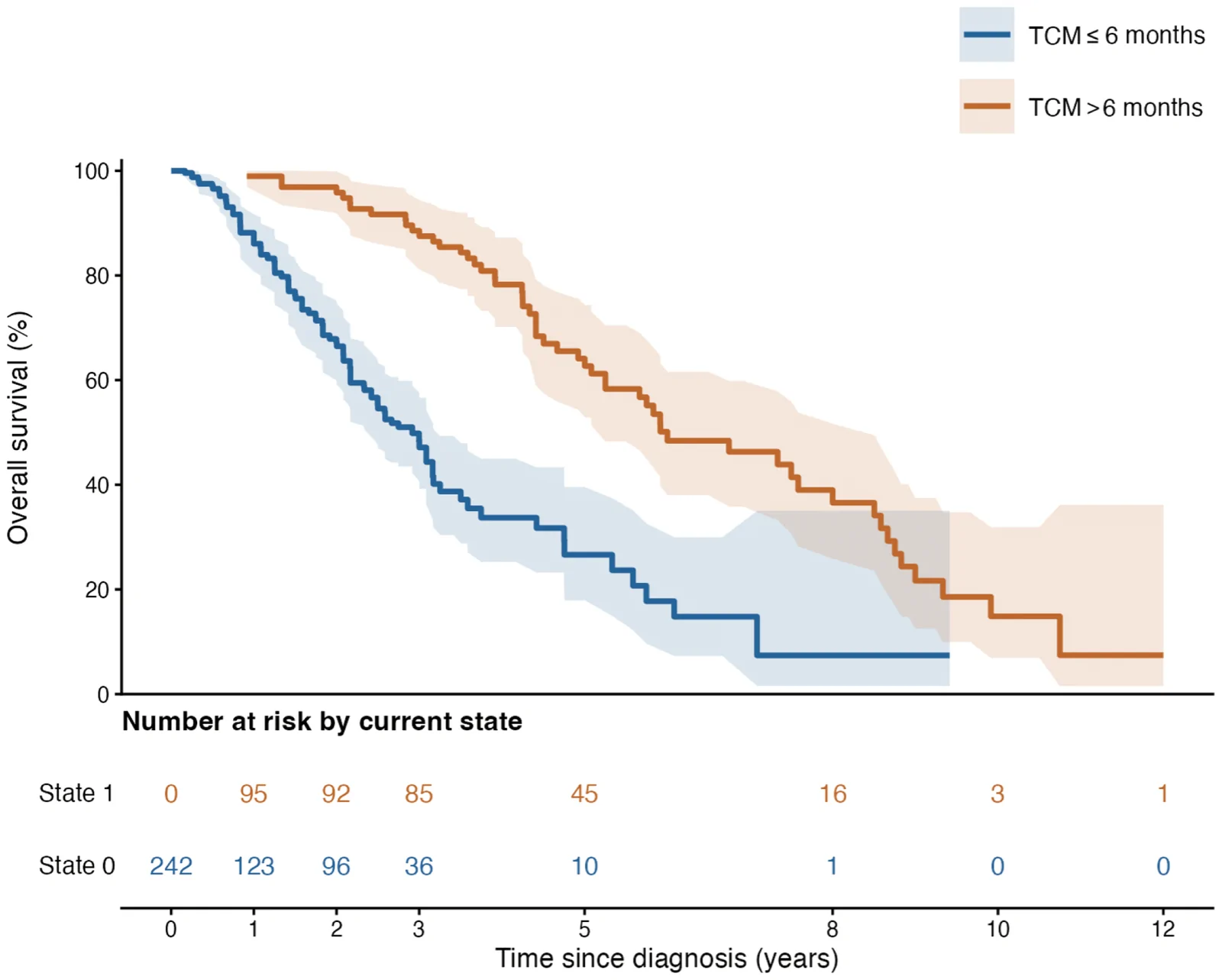
Simon–Makuch curves by time-dependent cumulative continuous TCM treatment status. All patients entered the cohort in the cumulative continuous TCM treatment ≤6-month state. Patients whose treatment exceeded 6 months transitioned to the >6-month state after reaching the threshold. Shaded areas represent 95% confidence intervals. The risk table shows the number of patients in each current exposure state at the specified times. Transitions from the ≤6-month to the >6-month state were not treated as loss to follow-up or terminal censoring. The curves are descriptive and were not used as the primary effect estimate. TCM, traditional Chinese medicine.

All patients entered the cohort in the cumulative continuous TCM treatment ≤6-month state. Patients whose treatment exceeded 6 months transitioned to the >6-month state after reaching the threshold. Shaded areas represent 95% confidence intervals. The risk table shows the number of patients in each current exposure state at the specified times. Transitions from the ≤6-month to the >6-month state were not treated as loss to follow-up or terminal censoring. The curves are descriptive and were not used as the primary effect estimate. TCM, traditional Chinese medicine.

### 3.6 Sensitivity and robustness analyses

When patients were classified according to their final recorded continuous TCM treatment duration, the unadjusted and adjusted HRs were 0.292 (95% CI, 0.198–0.430) and 0.257 (95% CI, 0.179–0.370), respectively. With the time-dependent exposure, the corresponding HRs were 0.306 (95% CI, 0.206–0.452) and 0.269 (95% CI, 0.186–0.389). The estimates were directionally consistent, and the adjusted time-dependent estimate was modestly closer to the null (**Supplementary Table S2**). The fixed classification was used only for methodological comparison.

All four prespecified sensitivity analyses produced estimates in the same direction as the primary model. After exclusion of 50 patients whose baseline PSA was recorded as an inexact threshold, the adjusted HR was 0.258 (95% CI, 0.171–0.389). Replacing CHAARTED tumor burden with LATITUDE risk classification or M1c status yielded adjusted HRs of 0.270 (95% CI, 0.187–0.389) and 0.257 (95% CI, 0.176–0.374), respectively. When the time-dependent exposure threshold was changed from 6 to 3 months, the adjusted HR was 0.341 (95% CI, 0.236–0.491). All P values were <0.001 (**Supplementary Table S3**).

Twelve potentially influential patients were identified using standardized DFBETA values. Removing any one of these patients produced adjusted HRs ranging from 0.251 to 0.279, with all P values <0.001. After cumulative exclusion of the one, three, and five most influential patients, the adjusted HRs were 0.251, 0.222, and 0.220, respectively. The direction of the association was unchanged in all influence analyses (**Supplementary Table S4**).

### 3.7 Sensitivity analyses addressing loss to follow-up

Forty-eight patients were lost to follow-up and right-censored on the last date they were confirmed alive. After excluding these patients, the adjusted HR for cumulative continuous TCM treatment exceeding 6 months was 0.211 (95% CI, 0.147–0.305; P<0.001). In inverse probability of censoring weighting (IPCW) analyses, the adjusted HRs were 0.270 (95% CI, 0.187–0.391) using untruncated weights and 0.269 (95% CI, 0.186–0.389) after truncation at the 1st and 99th percentiles. These estimates were close to the primary result, indicating that the association remained stable after adjustment for selective loss to follow-up explained by the measured baseline variables (**Supplementary Table S5**).

## 4 Discussion

In this single-center retrospective cohort, cumulative continuous TCM treatment exceeding 6 months was associated with a lower hazard of all-cause mortality among patients with metastatic prostate cancer (average adjusted HR, 0.269). Estimates remained in the same direction after changes to the exposure threshold, disease-severity adjustment, PSA handling, influential-patient exclusions, and approaches to loss to follow-up. These results support a consistent observational association between longer cumulative continuous TCM treatment and lower all-cause mortality risk.

This finding is broadly consistent with previous clinical evidence concerning adjunctive TCM in prostate cancer. A population-based retrospective cohort using Taiwan’s National Health Insurance Research Database reported an association between TCM use and improved survival among patients with metastatic prostate cancer (24), whereas a systematic review of randomized trials found limited evidence that oral Chinese herbal medicine combined with endocrine therapy might delay disease progression and improve survival or performance status (23). A recent review further summarized emerging experimental and clinical evidence for Chinese herbal medicines in prostate cancer management (20). The population-based cohort compared TCM users with nonusers, and the randomized-trial literature did not model cumulative treatment duration as a time-dependent exposure (23, 24). By examining treatment duration within a cohort in which all patients received TCM, the present study shifts the question from whether TCM was used to how cumulative continuous treatment duration was associated with survival. It thus adds a temporal dimension to the real-world evidence on long-term integrative management.

Several biological mechanisms could be consistent with the observed association, although the supporting evidence is predominantly preclinical (20–22). TCM treatment based on syndrome differentiation can be adjusted over time according to disease stage, functional status, and treatment response (18–20). Previous studies from our institution found that curcumin enhanced the growth-inhibitory effect of bicalutamide in prostate cancer cells and that polyphyllin I, alone or combined with enzalutamide, inhibited prostate cancer cells and xenograft growth (21, 22). These findings raise the possibility that TCM could contribute through direct antitumor activity, enhancement of antiandrogen treatment, or delayed treatment resistance. However, these mechanisms were not evaluated in the present cohort and cannot explain the observed survival association without further clinical and translational evidence.

The treatment-duration analysis also has methodological implications. Cumulative continuous TCM treatment exceeding 6 months was not a baseline characteristic; it emerged during follow-up. The time-dependent model allocated observation time before the threshold to the ≤6-month state and updated exposure only after the threshold was exceeded. The adjusted HR changed slightly from 0.257 in the fixed final-duration model to 0.269 in the time-dependent model after pre-threshold follow-up was allocated according to the patient’s current exposure state. The direction of the association was also retained when the threshold was reduced to 3 months. Six months was a prespecified analytical threshold rather than an established optimal treatment duration, and the clinically optimal duration cannot be inferred from these data.

This study has several limitations. First, its single-center retrospective design is vulnerable to selection bias, reverse causation, and residual confounding. Patients able to continue TCM may also have had better functional status, adherence, access to care, or other favorable characteristics that were not fully captured by the measured covariates. Second, because every patient received TCM, the study estimates the association between treatment duration and survival among TCM users; it does not estimate the effect of using TCM versus not using it. Third, continuous treatment from diagnosis was confirmed by the research team, whereas the database recorded total continuous treatment duration without prescription-level dates, treatment start and stop dates, or interruptions. This limits independent verification of the exposure timeline. Fourth, TCM prescriptions, doses, adherence, and concurrent conventional treatments were heterogeneous. The available data could not separate the effects of individual prescriptions or treatment combinations or fully address time-varying confounding by concurrent therapy. Fifth, 48 patients (19.8%) were lost to follow-up. Complete-case and IPCW estimates were close to the primary result, but IPCW can address only selective censoring explained by the measured variables. Finally, the study population was drawn from a single institution, limiting generalizability to other regions and care settings. Independent multicenter cohorts and prospective studies with detailed longitudinal treatment records are needed.

## 5 Conclusions

In this single-center retrospective cohort, cumulative continuous TCM treatment exceeding 6 months was associated with a lower hazard of all-cause mortality among patients with metastatic prostate cancer, and the direction of the association was consistent across prespecified sensitivity analyses. These findings provide real-world evidence on the temporal dimension of TCM treatment in long-term integrative management. Causality, the optimal treatment duration, and external applicability require evaluation in multicenter prospective studies.

## Data Availability

The original contributions presented in the study are included in the article/**Supplementary Material**. Further inquiries can be directed to the corresponding authors.
