# Supplementary material for: Impact of traditional Chinese medicine combined with conventional therapies on the survival of patients with metastatic prostate cancer: a retrospective observational cohort study
Source: Front Oncol. 2026 Oct 5;16:1948429. doi: 10.3389/fonc.2026.1948429 (PMC13677356; doi:10.3389/fonc.2026.1948429)
Supplement: Supplementary file 1 [file Supplementaryfile1.docx]

Supplementary Material

**Table S1. Time-varying association between cumulative continuous TCM treatment exceeding 6 months and all-cause mortality**

**Panel A. Interaction model coefficients**

| **Term** | **β estimate** | **Robust SE** | **P value** |
| --- | --- | --- | --- |
| Time-dependent cumulative continuous TCM treatment >6 months at 1 year | -2.325 | 0.519 | <0.001 |
| Time-dependent cumulative continuous TCM treatment >6 months × log(time/365) | 0.976 | 0.408 | 0.017 |

**Panel B. Estimated time-specific hazard ratios**

| **Follow-up time since diagnosis** | **Adjusted HR** | **95% CI** |
| --- | --- | --- |
| 1 year | 0.098 | 0.035–0.270 |
| 2 years | 0.192 | 0.110–0.335 |
| 3 years | 0.286 | 0.190–0.431 |
| 5 years | 0.471 | 0.274–0.807 |

n=242; deaths=147; 338 start–stop intervals. The model was adjusted for age at diagnosis, year of diagnosis, ECOG performance status, log₂-transformed baseline PSA lower bound, inexact PSA recording, Gleason score, and CHAARTED tumor burden. The model included an interaction between the time-dependent cumulative continuous TCM treatment exposure and log(time/365), with the exposure coefficient centered at 1 year. Time-specific adjusted HRs were calculated from the fitted interaction model. HRs below 1 indicate a lower estimated hazard associated with cumulative continuous TCM treatment exceeding 6 months at the specified time point. CI, confidence interval; ECOG, Eastern Cooperative Oncology Group; HR, hazard ratio; PSA, prostate-specific antigen; SE, standard error; TCM, traditional Chinese medicine.

**Table S2. Comparison of fixed and time-dependent modeling of cumulative continuous TCM treatment**

| **Model** | **HR** | **95% CI** | **P value** |
| --- | --- | --- | --- |
| Fixed final-duration classification, unadjusted | 0.292 | 0.198–0.430 | <0.001 |
| Fixed final-duration classification, adjusted | 0.257 | 0.179–0.370 | <0.001 |
| Time-dependent exposure, unadjusted | 0.306 | 0.206–0.452 | <0.001 |
| Time-dependent exposure, adjusted | 0.269 | 0.186–0.389 | <0.001 |

All analyses included 242 patients and 147 deaths. The fixed models classified patients according to their final recorded continuous TCM treatment duration (≤6 vs >6 months) and were conducted solely for methodological comparison. In the time-dependent models, all patients entered the cohort in the ≤6-month state, and eligible patients transitioned to the >6-month state upon exceeding 6 months of cumulative continuous TCM treatment, yielding 338 start–stop intervals. Adjusted models included age at diagnosis, year of diagnosis, ECOG performance status, log₂-transformed baseline PSA lower bound, an indicator for inexact PSA recording, Gleason score, and CHAARTED tumor burden. CI, confidence interval; ECOG, Eastern Cooperative Oncology Group; HR, hazard ratio; PSA, prostate-specific antigen; TCM, traditional Chinese medicine.

**Table S3. Sensitivity analyses of the association between cumulative continuous TCM treatment and all-cause mortality**

| **Analysis** | **Patients** | **Deaths** | **Start–stop intervals** | **Adjusted HR** | **95% CI** | **P value** |
| --- | --- | --- | --- | --- | --- | --- |
| Excluding patients with inexact baseline PSA records | 192 | 121 | 265 | 0.258 | 0.171–0.389 | <0.001 |
| Replacing CHAARTED tumor burden with LATITUDE risk classification | 242 | 147 | 338 | 0.270 | 0.187–0.389 | <0.001 |
| Replacing CHAARTED tumor burden with M1c status | 242 | 147 | 338 | 0.257 | 0.176–0.374 | <0.001 |
| Alternative exposure threshold: cumulative continuous TCM treatment >3 months | 242 | 147 | 383 | 0.341 | 0.236–0.491 | <0.001 |

Except for the specified modification, all sensitivity analyses used the same time-dependent Cox model structure and covariate adjustment as the primary analysis. The primary exposure threshold was cumulative continuous TCM treatment >6 months; in the alternative-threshold analysis, the exposure was defined as >3 months. The analysis excluding inexact baseline PSA records included only patients with exactly recorded PSA values. Two sensitivity models replaced CHAARTED tumor burden with either LATITUDE risk classification or M1c status, as specified. Tied event times were handled using the Efron method, and robust standard errors were clustered at the patient level. CI, confidence interval; HR, hazard ratio; PSA, prostate-specific antigen; TCM, traditional Chinese medicine.

**Table S4. Individual influence analyses for the association between cumulative continuous TCM treatment and all-cause mortality**

| **Analysis** | **Adjusted HR or range** | **95% CI or range** | **P value** |
| --- | --- | --- | --- |
| Leave-one-out analysis of 12 potentially influential patients | 0.251–0.279 | 0.172–0.403 | All <0.001 |
| Excluding the most influential patient | 0.251 | 0.172–0.366 | <0.001 |
| Cumulatively excluding the 3 most influential patients | 0.222 | 0.150–0.331 | <0.001 |
| Cumulatively excluding the 5 most influential patients | 0.220 | 0.148–0.326 | <0.001 |

Potentially influential patients were identified using patient-level standardized DFBETA values for the time-dependent TCM exposure coefficient, with a cutoff of 2/√N (N = 242; cutoff = 0.1286). Each leave-one-out model included 241 patients; sample sizes decreased accordingly in the cumulative-deletion analyses. The primary model was refitted after separately excluding each flagged patient and after cumulatively excluding the one, three, and five most influential patients. For the leave-one-out analysis, the reported HR range and confidence interval limits represent the ranges across the 12 refitted models. CI, confidence interval; HR, hazard ratio; TCM, traditional Chinese medicine.

**Table S5. Sensitivity analyses addressing loss to follow-up**

| **Analysis** | **Patients** | **Deaths** | **Adjusted HR** | **95% CI** | **P value** |
| --- | --- | --- | --- | --- | --- |
| Primary analysis | 242 | 147 | 0.269 | 0.186–0.389 | <0.001 |
| Excluding patients lost to follow-up | 194 | 147 | 0.211 | 0.147–0.305 | <0.001 |
| IPCW with untruncated stabilized weights | 242 | 147 | 0.270 | 0.187–0.391 | <0.001 |
| IPCW with stabilized weights truncated at the 1st and 99th percentiles | 242 | 147 | 0.269 | 0.186–0.389 | <0.001 |

Patients lost to follow-up were right-censored at the last date on which they were confirmed alive in the primary analysis. The censoring model used to derive the IPCW included age, year of diagnosis, ECOG performance status, log₂-transformed baseline PSA lower bound, inexact PSA recording, Gleason score, and CHAARTED tumor burden. Stabilized weights ranged from 0.741 to 1.589; truncated weights ranged from 0.802 to 1.291. CI, confidence interval; HR, hazard ratio; IPCW, inverse probability of censoring weighting; PSA, prostate-specific antigen; ECOG, Eastern Cooperative Oncology Group.
